# SA-4-1BBL Costimulation Inhibits Conversion of Conventional CD4^+^ T Cells into CD4^+^FoxP3^+^ T Regulatory Cells by Production of IFN-γ

**DOI:** 10.1371/journal.pone.0042459

**Published:** 2012-08-01

**Authors:** Shravan Madireddi, Rich-Henry Schabowsky, Abhishek K. Srivastava, Rajesh K. Sharma, Esma S. Yolcu, Haval Shirwan

**Affiliations:** Institute for Cellular Therapeutics, Department of Microbiology and Immunology, and James Brown Cancer Center, University of Louisville, Louisville, Kentucky, United States of America; Agency for Science, Technology and Research (A*STAR), Singapore

## Abstract

Tumors convert conventional CD4^+^ T cells into induced CD4^+^CD25^+^FoxP3^+^ T regulatory (iTreg) cells that serve as an effective means of immune evasion. Therefore, the blockade of conventional CD4^+^ T cell conversion into iTreg cells represents an attractive target for improving the efficacy of various immunotherapeutic approaches. Using a novel form of 4-1BBL molecule, SA-4-1BBL, we previously demonstrated that costimulation via 4-1BB receptor renders both CD4^+^and CD8^+^ T effector (Teff) cells refractory to inhibition by Treg cells and increased intratumoral Teff/Treg cell ratio that correlated with therapeutic efficacy in various preclinical tumor models. Building on these studies, we herein show for the first time, to our knowledge, that signaling through 4-1BB inhibits antigen- and TGF-β-driven conversion of naïve CD4^+^FoxP3^−^ T cells into iTreg cells via stimulation of IFN-γ production by CD4^+^FoxP3^−^ T cells. Importantly, treatment with SA-4-1BBL blocked the conversion of CD4^+^FoxP3^−^ T cells into Treg cells by EG.7 tumors. Taken together with our previous studies, these results show that 4-1BB signaling negatively modulate Treg cells by two distinct mechanisms: i) inhibiting the conversion of CD4^+^FoxP3^−^ T cells into iTreg cells and ii) endowing Teff cells refractory to inhibition by Treg cells. Given the dominant role of Treg cells in tumor immune evasion mechanisms, 4-1BB signaling represents an attractive target for favorably tipping the Teff:Treg balance toward Teff cells with important implications for cancer immunotherapy.

## Introduction

CD4^+^CD25^+^FoxP3^+^ Treg cells play a critical role in peripheral tolerance to self-antigens. As such, non-physiological alterations in their function or numbers are associated in immune abnormalities ranging from autoimmunity to cancer. In particular, a series of studies in preclinical as well as clinical settings have demonstrated the dominant role of Treg cells in cancer immune evasion mechanisms [1]. Treg cells accumulate within the tumor and in the secondary lymphoid organs as a result of tumor-mediated recruitment and/or expansion of preexisting natural Treg cells (nTreg cells) [2] or conversion of Teff cells into iTreg cells [3], [4]. Treg cells then suppress anti-tumor immune responses by targeting cells of innate, adaptive, and humoral immunity, thereby promoting tumor progression [1], [2]. Thus, Treg cells present an important therapeutic target for cancer immunotherapy. Consistent with this notion are studies demonstrating that physical depletion of Treg cells using antibodies to various cell surface markers or immunotoxins potentiates immunity to cancer with therapeutic consequences in various preclinical settings [1], [5], [6]. Although Treg cells were shown to accumulate in various tumors in the clinic and their presence serves as a significant negative prognostic factor [2], [7], physical depletion of Treg cells using antibodies or immunotoxins has resulted in varying outcomes ranging from lack of immune efficacy and clinical response to effective immunity and partial clinical response [8], [9]. The strikingly different outcomes seen between preclinical and clinical settings may be due to the nature of spontaneous tumors in the clinic vs. transplantable tumor in preclinical models, inefficiency of antibodies and immunotoxins to completely deplete Treg cells and their potential negative effect on Teff cells in the clinic [8], [9]. Therefore, alternative approaches that target effective inhibition of Treg cell generation/expansion during tumor progression and their physical and/or functional inactivation need to be developed for efficacy in the clinic.

Signaling through 4-1BB, a co-stimulatory molecule belonging to the TNF receptor family, plays an important role in the activation, proliferation, survival, and establishment of long-term memory of both CD4^+^ and CD8^+^ T cells [10], [11]. We, therefore, hypothesized that 4-1BB signaling can be exploited for the development of therapeutic vaccines and generated a chimeric molecule, SA-4-1BBL, with core streptavidin (SA) where the extracellular domain of the mouse 4-1BBL was fused C-terminus to SA [12], [13]. The SA portion of the molecule allows for oligomerization of the chimeric protein in soluble form that possesses pleiotropic effects on cells of innate, adaptive, and regulatory immunity, which translate into therapeutic efficacy in various preclinical tumor settings [13]. Importantly, we had previously demonstrated that SA-4-1BBL costimulation renders Teff cells refractory to suppression by Treg cells and increases the ratio of CD8^+^ Teff to Treg cells at the tumor site when used as the adjuvant component of tumor associated antigens (TAAs)-based vaccines [12], [13]. Given that cancer has evolved various mechanisms to effectively convert Teff cells into iTreg cells for immune evasion [3], [4], we hypothesized that 4-1BBL may prevent the conversion of Teff cells into iTreg cells in tumor settings, thereby resulting in a favorable Teff:Treg cell ratio and effective immunotherapy.

We herein tested this hypothesis by investigating the effect of immunomodulation with SA-4-1BBL on antigen-, TGF-β-, and tumor-mediated conversion of conventional CD4^+^ T cells into iTreg cells. To our knowledge, the studies presented in this manuscript demonstrate for the first time that SA-4-1BBL effectively inhibits antigen- and TGF-β-mediated conversion of CD4^+^FoxP3^−^ T cells into iTreg cells through induction of IFN-γ production by CD4^+^FoxP3^−^ T cells. Consistent with the *in vitro* data, immunomodulation with SA-4-1BBL blocks the conversion of conventional CD4^+^ T cells into iTreg cells in a tumor setting *in vivo*. Collectively, these studies demonstrate that 4-1BB signaling in conventional CD4^+^ T cells not only affect their activation, expansion, survival and establishment of long-term memory [14], but also renders these cells refractory to immunosuppression by Treg cells [13] as well as blocks their conversion into iTreg cells with significant potential for the development of therapeutic vaccines against cancer and chronic infections.

## Results

### SA-4-1BBL Inhibits Low Dose Antigen-mediated Conversion of Conventional CD4^+^ T Cells into CD4^+^CD25^+^FoxP3^+^ T Cells

**Figure 1:**
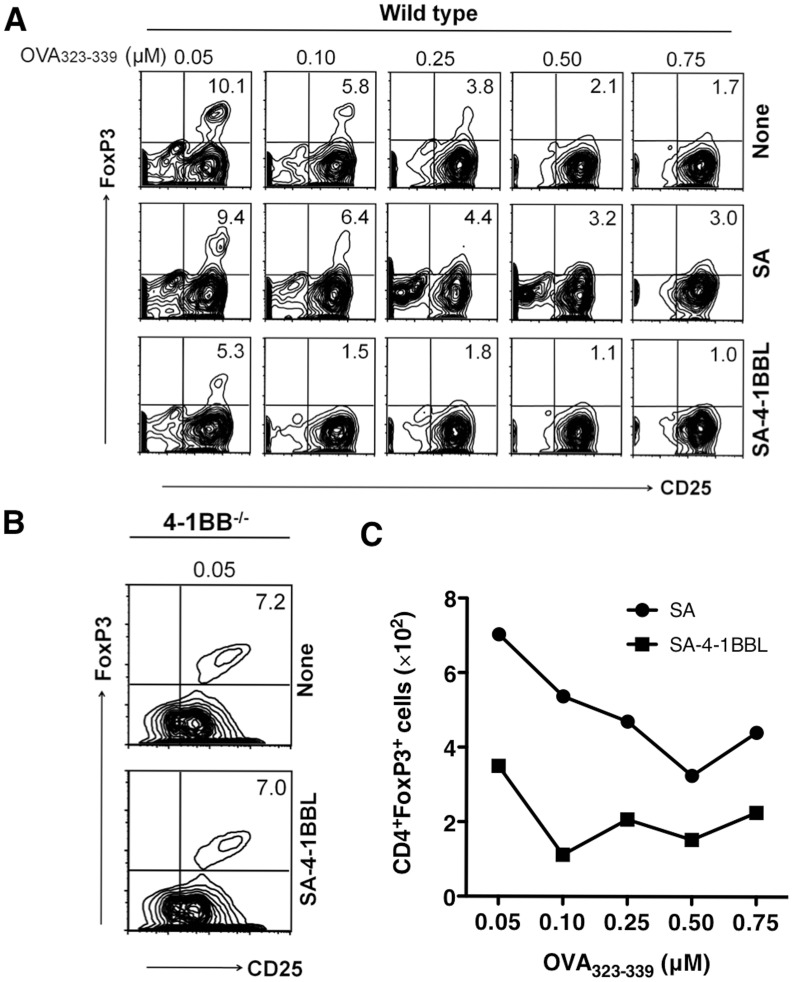
Signaling via 4-1BB inhibits antigen-mediated conversion of CD4^+^CD25^−^ T cells into CD4^+^CD25^+^FoxP3^+^ T cells. (A) Sorted CD4^+^ CD25^−^ T cells from naïve wild type OT-II transgenic mice were co-cultured with APCs, various doses of OVA_323–339_ peptide, and 10 µg/mL of SA-4-1BBL protein for 4 days in the absence of any exogenous cytokines. Untreated cultures (None) and those supplemented with SA protein served as controls. CD25^+^FoxP3^+^ cells were assessed by gating on CD4^+^ population using flow cytometry. Data is representative of at least three independent experiments. (B) as in (A), except CD4^+^ CD25^−^ T cells from naïve OT-II 4-1BB^−/−^ mice were used. (C) Absolute number of CD4^+^FoxP3^+^ T cells. Data are representative of three independent experiments.

Conventional CD4^+^ T cells were shown to convert into iTreg cells under low dose of antigenic stimulation [15]. This conversion process is known to depend on TGF-β signaling and conditions that prevent activation of APCs as well as IL-2 production by naïve T cells [15]. To test the effect of SA-4-1BBL on antigen driven conversion, naïve CD4^+^CD25^−^ OT-II T cells were co-cultured with T cell-depleted APCs and varying doses of OT-II epitope OVA_323–339_ peptide in the presence or absence of SA-4-1BBL with no exogenous TGF-β. There was an inverse correlation between the antigen dose and the percentage of CD4^+^CD25^+^FoxP3^+^ T cells with the lowest dose of 0.05 µM OVA_323–339_ peptide generating the most CD4^+^CD25^+^FoxP3^+^ T cells (Fig. 1A, upper row). Importantly, SA-4-1BBL significantly decreased the percentage (Fig. 1A, bottom row) and absolute number (Fig. 1C) of CD4^+^CD25^+^FoxP3^+^ T cells generated, predominantly at low dose antigenic stimulation. This effect was SA-4-1BBL specific and required 4-1BB signaling since wild type cells treated with SA protein (Fig. 1A, middle row) or *4-1BB^−/−^* cells treated with SA-4-1BBL (Fig. 1B) had similar levels of CD4^+^CD25^+^FoxP3^+^ T cells as compared with untreated controls.

As we used wild type APCs in coculture experiments with *4-1BB^−/−^* CD4^+^ T cells, the data presented in Fig. 1B further suggest that ligation of 4-1BB by SA-4-1BBL on CD4^+^ T cells, but not APCs, is required for the inhibition of conversion. To rule out a potential contribution of the anti-CD4 Ab used to isolate CD4^+^CD25^−^ OT-II T cells to the observed SA-4-1BBL-mediated inhibition of conversion, unsorted OT-II splenocytes were cultured with SA-4-1BBL and varying doses of OVA_323–339_ peptide. We observed a similar level of inhibition of conversion with unsorted OT-II cells as compared with the sorted CD4^+^CD25^−^ OT-II T cells (data not shown). Moreover, we did not observe conversion in cultures stimulated with SA-4-1BBL in the absence of peptide (data not shown), which is consistent with our published studies demonstrating that SA-4-1BBL has no costimulatory effect on naïve T cells [16]. Collectively, these data demonstrate that costimulation-mediated by SA-4-1BBL antagonizes the low dose antigenic stimulus required for the conversion of conventional CD4^+^ T cells into CD4^+^CD25^+^FoxP3^+^ T cells.

### SA-4-1BBL Inhibits TGF-β-mediated Conversion of Conventional CD4^+^ T Cells into CD4^+^CD25^+^FoxP3^+^ T Cells

**Figure 2:**
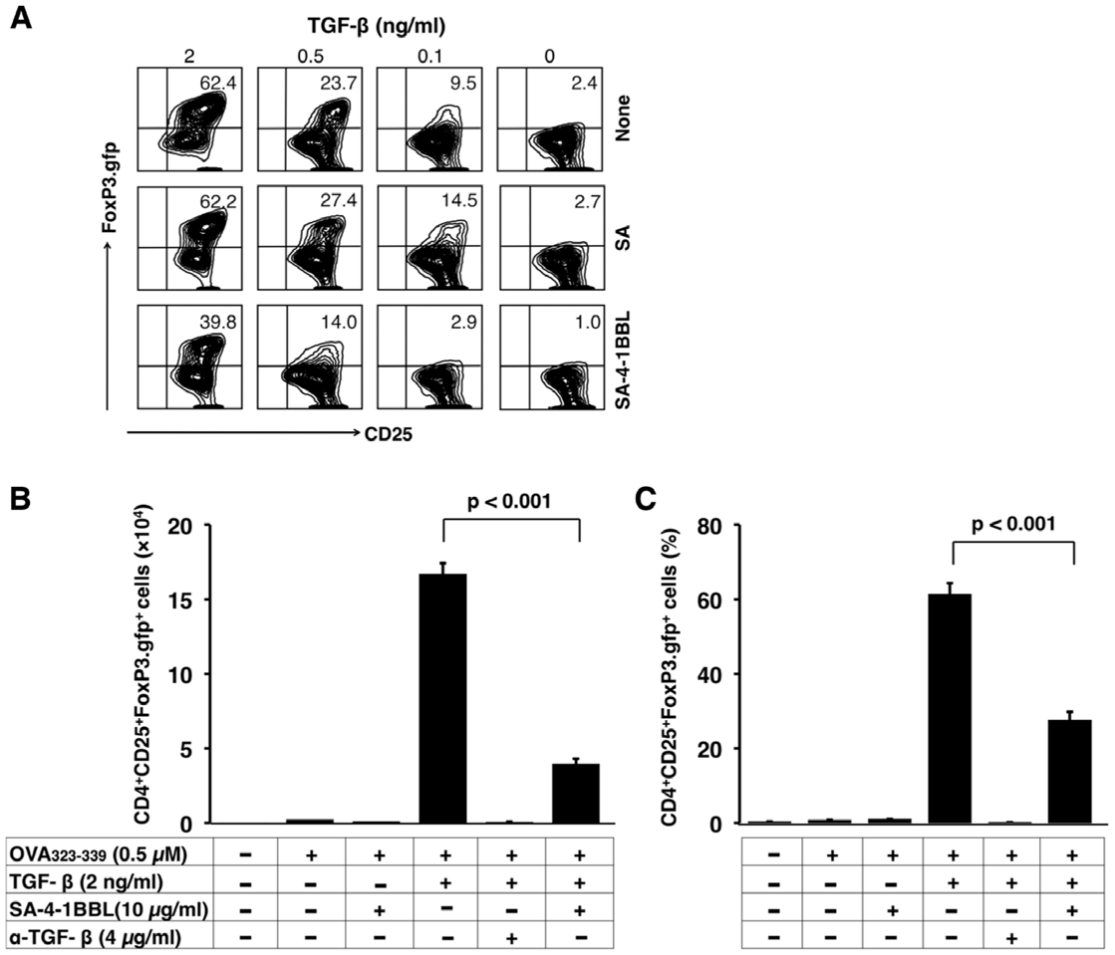
Signaling via 4-1BB inhibits TGF-β-mediated conversion of CD4^+^CD25^−^ T cells into CD4^+^CD25^+^FoxP3^+^ T cells. (A) Sorted OT-II CD4^+^FoxP3.gfp*^−^* T cells were cocultured with APCs, 0.5 µM OVA_323–339_ peptide, and various doses of exogenous TGF-β in the absence or presence of 10 µg/mL SA-4-1BBL or an equimolar concentration of SA protein as control. Live cells were phenotyped for CD25 and gfp (FoxP3) expression by gating on CD4^+^ T cells using flow cytometry. (B and C) as in (A), except some cultures were supplemented with a blocking Ab against TGF-β and analyzed for total cell number (B) or percentage of live CD4^+^FoxP3.gfp^+^ cells (C). Data are mean ± SEM and are representative of at least three independent experiments with similar results. *P* value was determined using Student’s *t* test.

TGF-β converts peripheral conventional CD4^+^ T cells into iTreg cells by inducing the expression of transcription factor FoxP3 [3], [15], [17], and this mechanism is extensively employed by tumors for immune evasion [18]. We, therefore, tested if costimulation by SA-4-1BBL inhibits the conversion of conventional CD4^+^ T cells into CD4^+^CD25^+^FoxP3^+^ T cells. Stimulation of naïve CD4^+^CD25^−^ FoxP3.gfp^−^ T cells at 0.5 µM OVA_323–339_ peptide dose in the presence of various doses of exogenous TGF-β resulted in the generation of CD4^+^CD25^+^FoxP3^+^ T cells in a dose dependent manner (Fig. 2A, upper row). A blocking Ab to TGF-β totally inhibited the generation of CD4^+^CD25^+^FoxP3^+^ T cells, demonstrating the specificity of TGF-β-mediated conversion (Fig. 2B and C). Consistent with the low-dose antigenic stimulation, SA-4-1BBL costimulation significantly inhibited the TGF-β-mediated conversion as assessed by percent as well as absolute numbers of CD4^+^ FoxP3.gfp^+^ T cells generated as compared with SA treated or untreated controls (Fig. 2). The inhibitory effect of SA-4-1BBL costimulation was Treg-specific and not due to a general inhibition/toxicity on total CD4^+^ T cell survival/proliferation as cultures treated with SA-4-1BBL had more cells than controls (data not shown).

### IFN-γ Induced by 4-1BB Signaling Inhibits the Conversion of Conventional CD4^+^ T Cells into CD4^+^CD25^+^FoxP3^+^ T Cells

**Figure 3:**
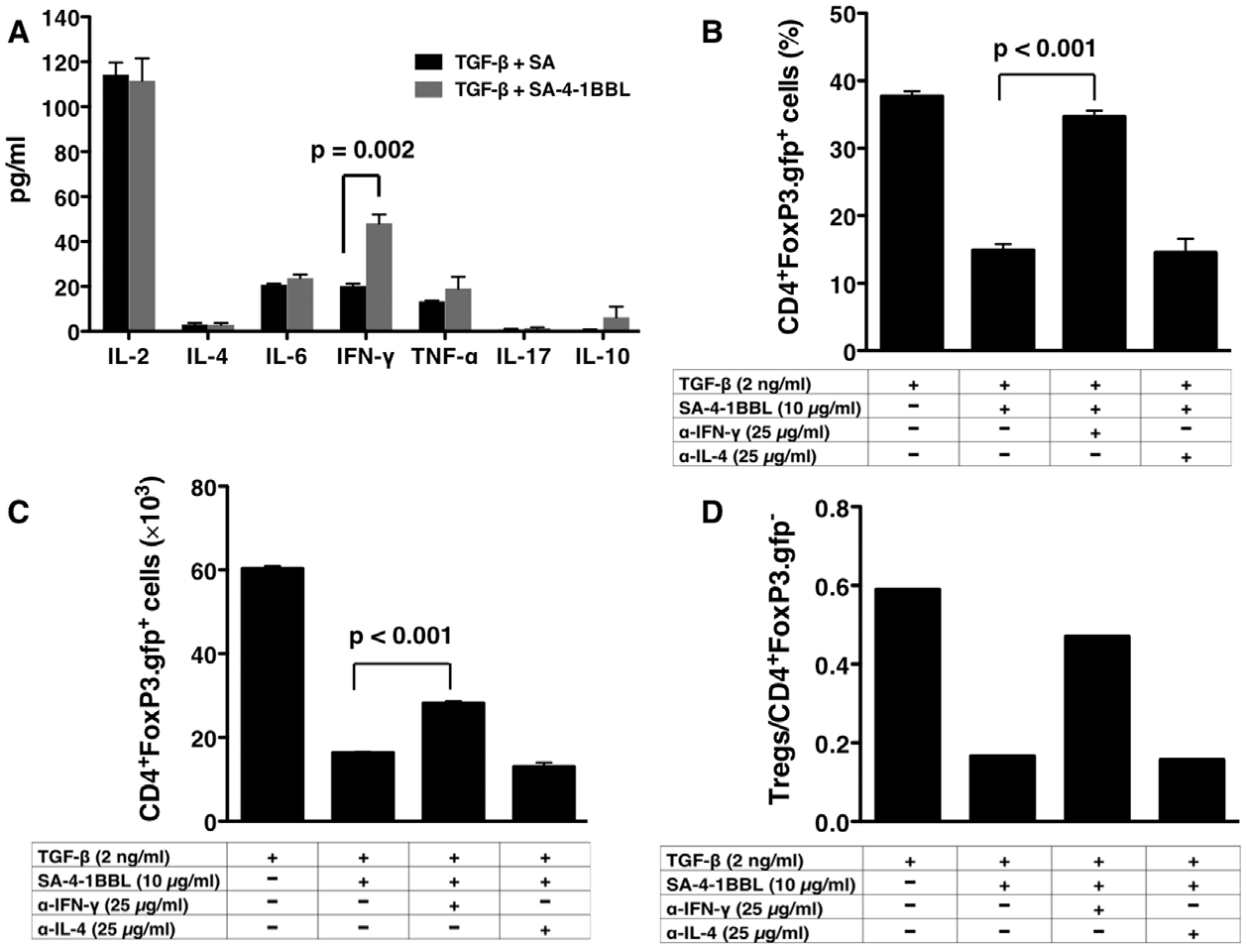
4-1BBL suppression of FoxP3 expression is mediated by IFN-γ. (A) CD4^+^FoxP3.gfp*^−^* T cells from naïve OT-II.FoxP3.gfp mice were cultured with APCs, 0.5 µM OVA_323–339_ peptide, and TGF- β (2 ng/mL) with or without 4-1BBL protein (10 µg/mL) for 4 days. Culture supernatants were analyzed for levels of various Th1/Th2/Th17 cytokines. (B, C and D) Cells were cultured as in (A), except cultures were supplemented with blocking Abs (25 µg/mL) to IFN-γ or IL-4. Live cells were analyzed by flow cytometry for CD25 and gfp (FoxP3) by gating on CD4^+^ T cells, and percentages (B), absolute numbers of CD4^+^FoxP3.gfp^+^ T cells (C), and ratio of CD4^+^FoxP3.gfp^+^ T cells/CD4^+^FoxP3^−^ T cells (D) were calculated. *P* value was determined using Student’s *t* test.

It has recently been demonstrated that signaling through OX40, another TNFR family costimulatory member, results in the inhibition of both low dose antigen as well as TGF-β-mediated conversion of naïve conventional CD4^+^ T cells into iTreg cells [19]. Both IFN-γ and IL-4 produced by activated CD4^+^ T cells were shown to act in synergy to block conversion. We, therefore, tested whether signaling through 4-1BB receptor in our model also exploits the same cytokines for the inhibition of CD4^+^CD25^−^ T cell conversion into CD4^+^CD25^+^FoxP3^+^ T cells. Analysis of culture supernatant from OT-II T cells stimulated with SA-4-1BBL/OVA_323–339_ peptide in the presence of TGF-β for a panel of cytokines demonstrated a significant increase only in the production of IFN-γ as compared with cultures without SA-4-1BBL stimulation (Fig. 3A). Although, there was production of IL-2, IL-6, and TNF-α, the levels of these cytokines were similar in cultures with and without SA-4-1BBL. Under these culture conditions, a blocking Ab against IFN-γ almost completely neutralized the SA-4-1BBL-mediated inhibition of CD4^+^CD25^−^ OT-II T cell conversion as assessed by the percentage of CD4^+^CD25^+^FoxP3^+^ T cells (Fig. 3B). However, this inhibition of conversion was significant (p<0.001), but not as pronounced when assessed by absolute number of CD4^+^CD25^+^FoxP3^+^ T cells recovered from cultures containing IFN-γ Ab/SA-4-1BBL/TGF-β as compared with TGF-β only control cultures (Fig. 3C). This discrepancy between the percentage and absolute number of CD4^+^CD25^+^FoxP3^+^ T cells appeared to be due to a negative effect of the IFN-γ Ab on the proliferation/expansion of both CD4^+^CD25^−^ OT-II and CD4^+^CD25^+^FoxP3^+^ T cell populations as assessed by the ratio of absolute cell numbers for these two populations (Fig. 3D). Regardless, we cannot rule out the contribution of another cellular/soluble factor in addition to IFN-γ to the observed SA-4-1BBL-mediated inhibition of conversion of CD4^+^CD25^−^ OT-II T to CD4^+^CD25^+^FoxP3^+^ T cells. A blocking Ab against IL-4 had no effect on the function of SA-4-1BBL in inhibiting iTreg cell generation. Collectively, these results demonstrate that signaling through 4-1BB receptor inhibits TGF-β-mediated conversion of conventional CD4^+^ T cells into CD4^+^CD25^+^FoxP3^+^ T cells and IFN-γ plays a critical role in this process.

### SA-4-1BBL Inhibits Tumor-mediated Conversion of Conventional CD4^+^ T Cells into CD4^+^CD25^+^FoxP3^+^ T Cells

**Figure 4:**
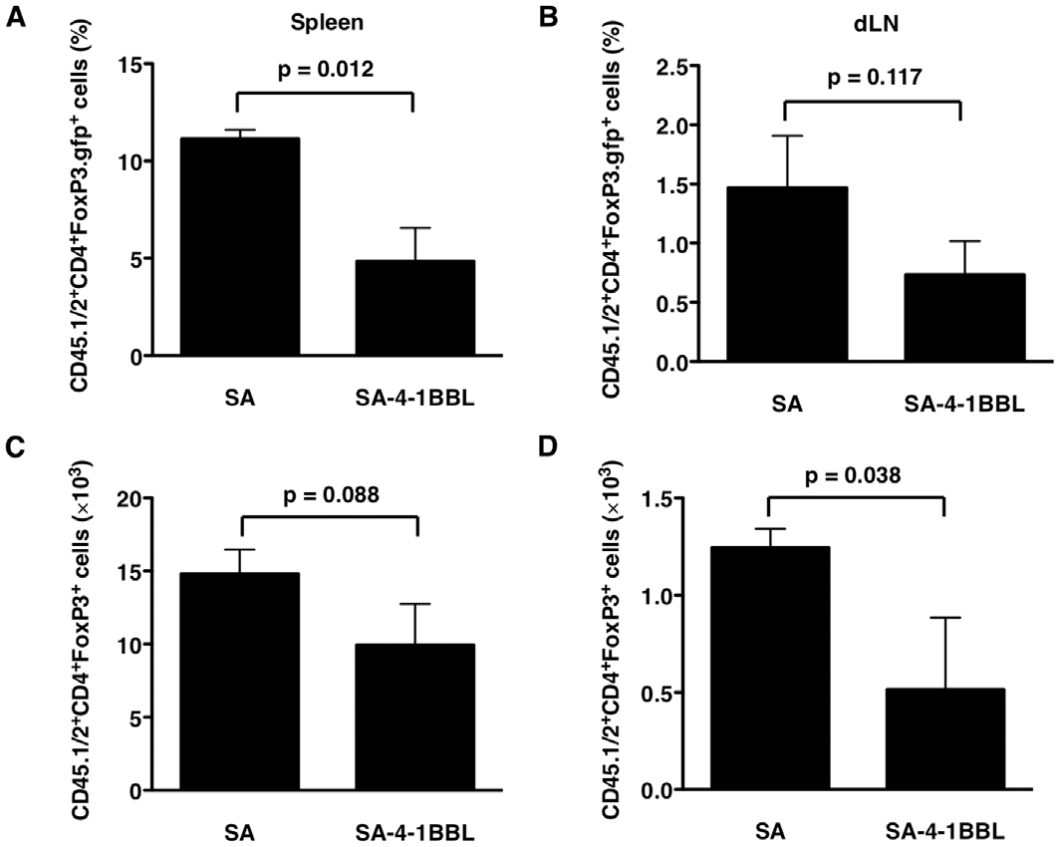
SA-4-1BBL blocks intratumoral conversion of CD4^+^FoxP3.gfp^−^ T cells into CD4^+^FoxP3.gfp^+^ T cells. C57BL/6.SJL (CD45.1) mice were challenged s.c. in the right flank with 2.5×10^6^ live OVA expressing EG.7 (CD45.2) tumor cells. (A and B) Sorted OT.II CD4^+^FoxP3.gfp*^−^* T cells (CD45.1/2) were injected intratumorally into tumor (9–10 mm in diameter) bearing C57BL/6.SJL (CD45.1) mice followed by intratumoral injection with SA-4-1BBL (25 µg) or an equimolar (10 µg) SA control protein one day later. The percentages (A, B) and absolute numbers (C, D) of CD45.1/2^+^CD4^+^FoxP3.gfp^+^ T cells in the spleen and draining LNs were assessed 5 days post treatment with SA-4-1BBL. (E) Absolute number of CD45.1/2^+^CD4^+^FoxP3.gfp^+^ T cells in the tumor tissues as assessed by flow cytometry. Data are representative of two independent experiments with at least 3 animals per group per experiment. (F) Confocal microscopic images of tumor tissues (100 µm upper and 50 µm lower panels) showing intracellular gfp (FoxP3, green) and cell nuclei (DAPI; blue). Tumor tissues from mice without OT.II CD4^+^FoxP3.gfp*^−^* T cell injection served as negative control for gfp signal. (G) Shows absolute number of cells (mean ± SEM) counted from confocal images of more than three areas/sample from three samples/group. *P* value was determined using Student’s *t* test.

Treg cells play a dominant role in tumor immune evasion mechanisms as demonstrated by a series of clinical and preclinical studies [1], [2], [4], [5], [6], [20]. Importantly, most tumors express high level of TGF-β, and as such actively convert tumor-specific Teff cells into iTreg cells as an effective means of immune evasion [18]. We, therefore, tested if immunomodulation with SA-4-1BBL affects the conversion of conventional CD4^+^ T cells into CD4^+^CD25^+^FoxP3^+^ T cells in the tumor microenvironment and peripheral lymphoid tissues. Sorted OT-II CD4^+^FoxP3.gfp^−^ cells were injected into established OVA expressing EG.7 tumors followed by treatment with SA-4-1BBL protein. There was a pronounced reduction in the percentage and absolute number of OT-II CD4^+^FoxP3.gfp^+^ T cells harvested from the spleen, tumor-draining lymph nodes, and tumor of mice treated with SA-4-1BBL as compared with untreated controls (Fig. 4A–D). Importantly, the most dramatic effect was seen in the tumor microenvironment as the absolute number of OT-II CD4^+^FoxP3.gfp^+^ T cells, assessed by flow cytometry (Fig. 4E) or confocal microscopy (Fig. 4F,G), were reduced in mice treated with SA-4-1BBL as compared with SA treated controls. This observation is consistent with our recently published studies demonstrating that immunization with a subunit vaccine consisting of SA-4-1BBL and human papilloma virus E7 TAA in a cervical cancer mouse model resulted in significant reduction in CD4^+^CD25^+^FoxP3^+^ Treg cells, which was correlated with robust therapeutic efficacy [12], [13]. Indeed, we observed significant retardation of EG.7 tumor growth during the 5-day short course experimental duration in mice treated with SA-4-1BBL as compared with controls (data not shown). Taken together, these results demonstrate that immunomodulation with SA-4-1BBL is effective in preventing the conversion of conventional CD4^+^ T cells into CD4^+^CD25^+^FoxP3^+^ T cells in a tumor microenvironment.

## Discussion

The 4-1BB/4-1BBL system has emerged as an important immunoregulatory pathway [21]. Signaling through 4-1BB receptor plays a critical role in modulating not only adaptive, but also innate and regulatory immunity [11], [21], [22], [23], [24]. As such, modulation of this pathway may have important therapeutic implications in settings of autoimmunity, cancer, and chronic infections. In the context of regulatory immunity, signaling via 4-1BB receptor may have diverse and opposing consequences. For example, we [25] and others [23], [24] have previously demonstrated that signaling via 4-1BB in nTreg cells in the presence of exogenous IL-2 results in their survival and expansion. However, Treg cells have no suppressive function in the presence of anti-receptor agonistic Abs or 4-1BBL. This is because signaling via 4-1BB receptor on Teff cells renders them refractory to inhibition by Treg cells via undefined mechanisms [13], [22]. Inasmuch as 4-1BB signaling primarily affects the expansion, survival, and function of Teff cells [14], the physiological consequence of co-expansion of both Teff and Treg cells via 4-1BB signaling remains to be determined. It is likely that the Treg cells co-expanded with Teff cells during the course of immune response to infections may serves as an alternative mechanism to activation induced cell death to achieve immune homeostasis by controlling the function of Teff cells once infection is cleared, thereby minimizing the collateral damage.

The present study reveals another important and novel function of 4-1BB signaling on regulating the balance between adaptive and regulatory immunity, i.e. blocking the de novo generation of iTreg cells from conventional CD4^+^ T cells through IFN-γ production. This observation is consistent with a recent study demonstrating that the low-dose antigen- or TGF-β-mediated conversion of conventional CD4^+^ T cells into iTreg cells is regulated by Th1 and Th2 cytokines, IFN-γ and IL-4, through transcriptional factors T-bet and GATA-3, respectively [26]. *In vitro* Ab blockade of IFN-γ resulted in TGF-β-driven expression of FoxP3 in naïve conventional CD4^+^ T cells. Importantly, forced expression of T-bet in T cells was sufficient to block FoxP3 expression in a cell autonomous fashion, demonstrating the critical role of this transcription factor in regulating the choice of naive conventional CD4^+^ T cells towards Th1, but not Treg, lineage commitment. These results are also consistent with the findings that CD4^+^ T cells with a CD44^hi^ memory and effector phenotype actively inhibit the TGF-β-induced conversion by producing IL-4, IL-21, and IFN-γ [27], suggesting a notion that inducer (such as 4-1BB signaling) or inhibitors (such as retinoic acid) of these Th1 cytokines can fine tune the immune cells by shifting the balance towards either effector or regulatory immunity, respectively.

These features are not unique to 4-1BB as two other members of TNFR superfamily, OX-40 and glucocorticoid-induced TNF receptor-related protein (GITR), have similar effects in regulating the balance between Teff and Treg cells. Signaling through both OX-40 and GITR receptors was shown to result in the proliferation and survival of both Treg and Teff cells as well as render Teff cells refractory to suppression by Treg cells [28], [29]. Although it was initially suggested that GITR signaling in Treg cells is critical for counteracting their suppressive activity [28], later studies using Teff and Treg cells from GITR deficient mice demonstrated the opposite [29]. In contrast to 4-1BB and GITR, OX-40 signaling directly blocks the inhibitory function of Treg cells via the down regulation of FoxP3 expression [30], [31], [32]. Similar to 4-1BB, OX-40 signaling also utilizes IFN-γ either alone or in combination with IL-4 or IL-6 to block the conversion of conventional CD4^+^ T cells into iTreg cells [19], [33]. Given the preferential effect of signaling via OX-40 on CD4^+^ and 4-1BB on CD8^+^ T cells [34] and the importance of both of these cell types for the generation of effective immune responses, a combinatorial use of agonists for these two receptors bears significant therapeutic potential for cancer and chronic infections.

The observation that signaling via 4-1BB prevents antigen-, TGF-β, and tumor-mediated conversion of conventional CD4^+^ T cells into iTreg cells via IFN-γ is consistent with and provide a plausible mechanistic insight into our recently published studies demonstrating that vaccination with SA-4-1BBL and human papilloma virus E7 TAA showed robust therapeutic efficacy against established TC-1 tumors [12], [13], [35]. The vaccine therapeutic efficacy was associated with tumor-specific killing activity, high frequency of IFN-γ secreting CD4^+^ T and CD8^+^ T cells, increased intratumoral number of CD4^+^ T and CD8^+^ T cells, and a decrease in the number of CD4^+^FoxP3^+^ Treg cells [12], [13], [35]. It is, therefore, tempting to speculate that the reduced number of OT-II CD4^+^FoxP3.gfp^+^ T cells observed in the EG.7 tumor in the present study may be due to the ability of SA-4-1BBL to stimulate conventional CD4^+^ and CD8^+^ T cells for the production of IFN-γ, which in turn blocks the conversion of CD4^+^ T cells into iTreg cells.

Moreover, the SA-4-1BBL used in our studies show no detectable toxicity in contrast to toxic effects reported with various anti-4-1BB agonistic Ab therapies [16], [36], [37]. Indeed, in comparative studies, we recently demonstrated that SA-4-1BBL had qualitative and quantitative differences than an agonistic 4-1BB Ab (3H3) in inducing immune responses, which resulted in better therapeutic efficacy against tumors in the absence of agonistic Ab-induced severe toxicity [13], [16]. Therefore, the triple function of 4-1BB signaling on T cells to; i) enhance activation of naïve T cells, survival, acquisition of effector function, and differentiation into long-term memory with a Th1 phenotype [11], [13], [21], [38], ii) render Teff cells refractory to inhibition by Treg cells [13], and iii) block the conversion of conventional CD4^+^ T cells into iTreg cells, reported in the present study, combined with lack of toxicity, may have important implications for the treatment of cancer and chronic infections that exploit Treg cells for immune evasion [1], [2], [5], [6], [20], [30] and require a Th1 dominated response for immunotherapy [12], [13], [21], [38].

## Materials and Methods

### Mice

OVA-specific TCR-transgenic C57BL/6 OT-II (*Rag^−/−^*) mice were purchased from Taconic Farms. Transgenic FoxP3.gfp (B6.Cg-FoxP3^tm2(EGFP)Tch^/J-Stock number 006772) and C57BL/6 mice were purchased from The Jackson Laboratory. OT-II.FoxP3.gfp mice were generated by crossing B6.Cg-FoxP3^tm2(EGFP)Tch^/J with OT-II (*Rag^−/−^*) mice. OT-II.FoxP3.gfp (CD45.1/2) mice were generated by crossing C57BL/6.FoxP3.gfp (CD45.2) with OT-II.SJL (CD45.1) mice. OT-II *4-1BB^−/−^* mice were generated by crossing C57BL/6 *4-1BB^−/−^* [39] with OT-II (*Rag^−/−^*) mice.

### CD4^+^ T Cell Sorting and APC Preparation

CD4^+^ FoxP3.gfp^−^ T cells were sorted from spleen and lymph nodes of OT-II.FoxP3.gfp mice using FACSAria (BD Biosciences) [13]. In experiments where OT-II (*Rag^−/−^*) and OT-II 4-1BB^−/−^ mice were used, we sorted CD4^+^ CD25^−^ T cells, due to unavailability of FoxP3.gfp background on these mice. The sorted cells were >98% pure. T cell depleted splenocytes were used as an enriched source of APCs. These were prepared by incubating naïve C57BL/6 splenocytes with an anti-αβ-TCR Ab (clone H57–597) and Low-Tox-M rabbit complement (Cedarlane Laboratories). After several washes with PBS, the live cells (>98% negative for T cells) were irradiated with 2,000 cGy and used as APCs in coculture experiments with sorted T cells [13].

### Conversion of Conventional CD4^+^ T Cells into FoxP3^+^ iTreg Cells *In vitro* and Cytokine Analysis

Sorted CD4^+^CD25^−^ T cells (0.5×10^6^ cells/mL) were cocultured with APCs (2×10^6^ cells/mL) in complete MLR medium supplemented with 0.05–0.5 µM OVA_323–339_ peptide for 4 days. Cultures were also supplemented with varying amounts (0–2 ng/mL) of recombinant human TGF-β1 (PeproTech, Rocky Hill, NJ) and 10 µg/mL of SA-4-1BBL or an equimolar concentration of SA protein as control. SA-4-1BBL and SA control proteins were produced using S2 cells with undetectable endotoxin levels [12]. Cells were stained with Abs to CD4-APC (Clone RM4–5) or CD4-PE (Clone GK1.5), CD25-PE or CD25 PE-Cy7 (Clone PC61), and intracellular FoxP3-PE according to the manufacturer’s protocol (eBioscience). Isotype Abs with matched fluorochromes were used as controls. All samples were collected using FACSCalibur or BD LSR II (BD Biosciences) and analyzed using FlowJo (Tree Star) or FACSDiva software.

Culture supernatants were collected and analyzed for cytokines using a BD Cytometric Bead Array (CBA) for mouse Th1/Th2/Th17 cytokines and analyzed using FCAP Array software (Soft Flow, Inc.).

### Conversion of Conventional CD4^+^ T Cells into iTreg Cells in EG.7 Tumor Model

C57BL/6.SJL (CD45.1) mice were challenged s.c. in the right flank with 2.5×10^6^ live OVA expressing EG.7 (CD45.2) tumor cells. When the tumor size reached 9–10 mm in diameter, 4×10^6^ sorted CD4^+^FoxP3.gfp^−^ cells from OT-II.FoxP3.gfp (CD45.1/2) mice were adoptively transferred by intratumoral injection. Mice were vaccinated one day later intratumorally with 25 µg SA-4-1BBL or equimolar (10 µg) SA control protein. Spleen, draining LNs, and tumor were harvested 5 days later and processed into single cell suspension as described [13]. Cells were stained with CD45.1-PE, CD45.2-APC, CD4-Alexa 700 and CD25-PE-Cy7 and analyzed using flow cytometry. Confocal microscopy for gfp (FoxP3) was performed on tumor tissues processed and fixed as published [12]. Five micron sections were stained with DAPI and analyzed for DAPI and gfp [12], [13].

### Statistical Analysis

Data were analyzed using Student’s *t-*test and were expressed as means ± SEM. *P<*0.05 was considered significant.
