# Correction: SA-4-1BBL Costimulation Inhibits Conversion of Conventional CD4^+^ T Cells into CD4^+^FoxP3^+^ T Regulatory Cells by Production of IFN-γ

**DOI:** 10.1371/annotation/88b557df-1e27-4a60-8b47-214a5cc3c707

**Published:** 2012-08-09

**Authors:** Shravan Madireddi, Rich-Henry Schabowsky, Abhishek K. Srivastava, Rajesh K. Sharma, Esma S. Yolcu, Haval Shirwan

Sections E,F, and G of Figure 4 do not appear. Please view the image for Figure 4E-G here: 

**Figure:**
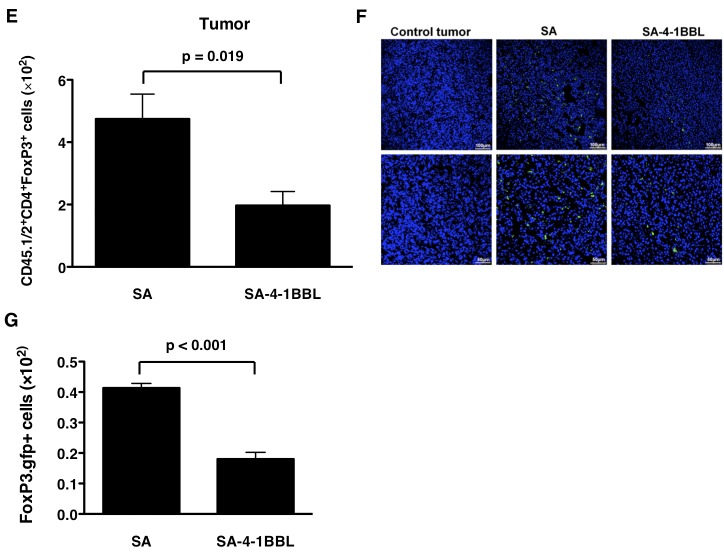


[^] 

